# Family-Centric Applied Behavior Analysis Facilitates Improved Treatment Utilization and Outcomes

**DOI:** 10.3390/jcm13082409

**Published:** 2024-04-20

**Authors:** Robert P. Adelson, Madalina Ciobanu, Anurag Garikipati, Natalie J. Castell, Navan Preet Singh, Gina Barnes, Jodi Kim Rumph, Qingqing Mao, Henry S. Roane, Anshu Vaish, Ritankar Das

**Affiliations:** 1 Montera, Inc., dba Forta, Research and Development, 548 Market St., PMB 89605, San Francisco, CA 94104-5401, USA; robert.adelson@fortahealth.com (R.P.A.); mciobanu@fortahealth.com (M.C.); agarikipati@fortahealth.com (A.G.); natalie.castell@fortahealth.com (N.J.C.); nsingh@fortahealth.com (N.P.S.); gbarnes@fortahealth.com (G.B.); anshu@fortahealth.com (A.V.); ritankar@fortahealth.com (R.D.); 2 Madison-Irving Medical Center, Upstate Medical University, 475 Irving Avenue, Syracuse, NY 13210-1756, USA; roaneh@upstate.edu

**Keywords:** applied behavior analysis, autism spectrum disorder, disruptive behavior, telehealth, treatment outcomes

## Abstract

**Background/Objective:** Autism spectrum disorder (ASD) is a neurodevelopmental condition characterized by lifelong impacts on functional social and daily living skills, and restricted, repetitive behaviors (RRBs). Applied behavior analysis (ABA), the gold-standard treatment for ASD, has been extensively validated. ABA access is hindered by limited availability of qualified professionals and logistical and financial barriers. Scientifically validated, parent-led ABA can fill the accessibility gap by overcoming treatment barriers. This retrospective cohort study examines how our ABA treatment model, utilizing parent behavior technicians (pBTs) to deliver ABA, impacts adaptive behaviors and interfering behaviors (IBs) in a cohort of children on the autism spectrum with varying ASD severity levels, and with or without clinically significant IBs. **Methods:** Clinical outcomes of 36 patients ages 3–15 years were assessed using longitudinal changes in Vineland-3 after 3+ months of pBT-delivered ABA treatment. **Results:** Within the pBT model, our patients demonstrated clinically significant improvements in Vineland-3 Composite, domain, and subdomain scores, and utilization was higher in severe ASD. pBTs utilized more prescribed ABA when children initiated treatment with clinically significant IBs, and these children also showed greater gains in their Composite scores. Study limitations include sample size, inter-rater reliability, potential assessment metric bias and schedule variability, and confounding intrinsic or extrinsic factors. **Conclusion:** Overall, our pBT model facilitated high treatment utilization and showed robust effectiveness, achieving improved adaptive behaviors and reduced IBs when compared to conventional ABA delivery. The pBT model is a strong contender to fill the widening treatment accessibility gap and represents a powerful tool for addressing systemic problems in ABA treatment delivery.

## 1. Introduction

Autism spectrum disorder (ASD) is a neurodevelopmental condition characterized by social communication impairments and restricted, repetitive behaviors (RRBs) that persist into adulthood [1]. The manifestation of ASD is highly variable, but many individuals experience deficits in executive functioning and daily living skills, difficulties with forming and maintaining social relationships, and negative responses to external stimuli [2]. Screening for ASD typically occurs in pediatric primary care settings and, while some health systems report success with broad screening of pediatric populations (e.g., >91% of children were screened at one hospital), variable performance of ASD screening assessments may lead to missed diagnoses [3]. The Diagnostic and Statistical Manual of Mental Disorders, Fifth Edition (DSM-5) classifies ASD within three severity levels, wherein an individual “requiring support” would be diagnosed with mild ASD, “requiring substantial support” with moderate ASD, and “requiring very substantial support” with severe ASD [4]. Research on the etiology of ASD is constantly evolving, and there are many questions that remain unanswered. Some recent research suggests that ASD may be, for some individuals, attributed to inherited genetic traits or genetic mutations [5], or adverse environmental exposures [6]. ASD is estimated to occur in 1 out of 36 children of eight years of age in the US, with a growing prevalence [7,8,9,10]. Its impact can be ameliorated by timely delivery of validated interventions, and thus access to treatment is imperative [11,12,13].

Applied behavior analysis (ABA), the gold-standard treatment for ASD, is supported by substantial research demonstrating effectiveness in improving outcomes [12,14,15,16]. ABA improves social, language, and communication skills, and reduces interfering behaviors (IBs) [15,17,18]. IBs—or maladaptive/challenging behaviors—are broadly defined as behaviors that can be harmful, impair daily functioning, or interfere with achieving patient/family goals [19,20,21]. IBs prevent constructive coping techniques and can endanger safety. ABA success relies on personalized treatment plans (TPs) that target skill acquisition and IB reduction; TPs are developed by board-certified behavioral analysts (BCBAs) after assessing skills and behaviors, and are implemented by behavior technicians (BTs) who received 40 hours of ABA training and are overseen by BCBAs [22,23,24]. Pharmacological treatments are also used for some individuals diagnosed with ASD to mitigate the impact of comorbidities, such as concurrent psychological disorders [2].

There are limitations to implementing ABA broadly, and families must overcome many logistical hurdles to access the necessary treatment [25]. Some barriers include shortages of qualified ABA providers [26], travel time to reach available clinics, treatment waitlists, and other scheduling issues [27]. In rural areas, these problems are exacerbated by low patient density, which can be inadequate to support local providers [27]. These challenges also contribute to treatment cost, which can be prohibitive; it is estimated that most families pay between $17,000 and $130,000 USD/year for ABA [27,28]. Telemedicine has emerged as a potential tool to mitigate these issues, but it fails to address the inadequate number of BTs, given that some of the limitations faced by BTs with telehealth reside in challenges detecting behaviors or responses in a patient [29].

ABA principles implemented by trained caregivers have emerged as a partial solution to treatment accessibility limitations, and the effectiveness of caregiver-implemented ABA has been repeatedly demonstrated for ASD [17,18,25,27,30]. Gerow et al. (2023) evaluated a telehealth program in which caregivers were trained and engaged in ABA methods with their child to assess changes across multiple domains, as measured by Vineland Adaptive Behavior Scales, Third Edition (Vineland-3); a significant effect size was observed for >50% or patients within the domains of daily living skills, socialization, and challenging behaviors (IBs) [18]. Sneed et al. (2022) demonstrated the value of caregiver participation in ABA, indicated by reduced parental stress with improved self-efficacy resulting from caregiver-led ABA [17], which can impact treatment outcomes [31]. Buzhardt et al. (2016) examined generalizability of an online ABA training program for Hispanic families with a child on the autism spectrum by incorporating a Spanish-language training program, the use of an interpreter, and culturally adapted ABA practices, yielding caregiver knowledge and skill gains for ABA implementation [32]. Bordini et al. (2024) examined outcomes for children on the autism spectrum aged 3–7 years whose caregivers were trained in ABA methods via instructional videos to provide prompts to elicit social communication behaviors (eye contact and joint attention) from the child [33]. Statistically significant changes in Vineland scores associated with joint attention were observed, demonstrating an increase in joint attention behaviors with less prompting over time [33]. Ferguson et al. (2022) examined telehealth-based ABA coaching for caregivers for use in playtime scenarios to improve manding and the ability of the child to establish eye contact with the caregiver, demonstrating variable increases among participants for increased eye contact, but minimal improvement in manding [34]. Abdi et al. (2023) examined caregiver-delivered pivotal response treatment, which utilities ABA techniques, as a supplement to routine ABA for development of social skills in children on the autism spectrum aged 2–6 years [35]. A control group received only clinic-based ABA without any supplemental involvement of caregivers. Statistically significant increases in the Autism Treatment Evaluation Checklist (ATEC) scores for socialization within the experimental group were observed, indicating improved social skills not observed in the control group [35].

The proximity of caregivers to their children can offset logistical challenges and provide access to ABA methodologies in on-demand, naturalistic/everyday situations [36]. However, caregiver-implemented ABA suffers from limitations that can hinder utility, including poor parental adherence, lack of consistency in applying ABA concepts, inadequate training support by ABA-trained clinicians (e.g., BCBAs), lack of comprehensive—if any—clinical oversight post-training (i.e., over the course of ABA implementation), and struggles with insurance reimbursement [37,38,39,40]. Overcoming these limitations would allow patients to benefit from much-needed treatment.

A sustainable caregiver/parent ABA treatment model must rely heavily on caregivers/parents (hereinafter, parents) for treatment delivery and implementation of ABA concepts. Parents must (1) be adequately trained as BTs, and (2) deliver treatment under the close supervision of highly qualified clinicians (i.e., at least 5% treatment oversight time by a BCBA [22]). Additionally, the treatment framework needs to promote consistency of scheduling with high treatment fidelity, high utilization of prescribed ABA treatment, and family buy-in, while eliminating barriers created by waitlists/provider availability, scheduling, transportation/remote geography, and cost [25,27,30]. To increase access to formalized and consistent treatment, Montera, Inc. dba Forta (hereinafter, Forta), an ABA provider company, draws upon the capabilities and proximity of parents to their children on the autism spectrum, which affords the unique opportunity to deliver scheduled treatment as well as on-demand, naturalistic ABA.

To empower parents to deliver ABA and mitigate treatment inaccessibility, Forta has developed a parent BT (pBT) model of ABA treatment (hereinafter, pBT Forta model or pBT model), which utilizes a framework of family buy-in, rigorous parent training, post-training assessment, and ongoing, consistent treatment supervision and feedback from BCBAs. The pBT model provides equitable access to ABA, regardless of geography, provider availability, or insurance type. It further benefits from extensive operational support from scheduling, technology, and billing teams, the latter of which act as a liaison between parents and their insurers. pBTs are provided with proprietary technology-based tools, such as an in-house-built software application (hereinafter, app) that streamlines treatment recording and monitoring, as well as an extensive library of ASD educational materials developed by Forta for pBT use. The pBT model intrinsically facilitates treatment consistency and high utilization of prescribed ABA treatment, while leveraging parents’ unparalleled knowledge of their children’s goals, potential, and struggles. We have previously reported on the initial 90+ day clinical outcomes of a cohort of children receiving treatment via the pBT model, yielding patient growth in skill acquisition goals across multiple domains [41].

This follow-up investigation examines the relationship between utilization of prescribed treatment and clinical outcomes for patients receiving ABA treatment within the pBT Forta model, as measured by the Vineland-3 [42]. Patient outcomes in the pBT model were analyzed using a retrospective cohort approach and were compared with Vineland-3 data from peer-reviewed studies [18,43,44]. We primarily hypothesized that increased treatment utilization would correlate with improved clinical outcomes. Additionally, we used a retrospective cohort study to address our secondary hypothesis: that patients initiating treatment with clinically significant IBs would have higher treatment utilization, and thus greater clinical improvements, compared to patients starting treatment without clinically significant IBs.

## 2. Materials and Methods

### 2.1. Study Design

The retrospective patient ABA treatment data used in this investigation were collected as part of our standard recordkeeping practices during ABA treatment, and were de-identified prior to analysis in compliance with HIPAA and in accordance with the 1964 Helsinki Declaration, revised in 2013. The protocol was approved with an exemption determination per 21CFR56.104 and 45CFR46.104(b)(4), and received a waiver of informed consent by the ethics committee of Pearl IRB (Indianapolis, IN, USA; protocol number 22-MONT-102).

To evaluate the efficacy of the pBT model on child behavior changes within multiple domains and subdomains, we compared the clinical outcomes of Forta’s patients with previously published data from studies on conventional and caregiver-implemented ABA that measured patient behavior changes using Vineland-3 scores (Pearson Assessments, San Antonio, TX, USA) [18,43,44,45]. This aligns with how Forta’s (San Francisco, CA, USA) BCBAs measure patient progress and behavior changes: Vineland-3 assessments are given as clinically indicated within the course of treatment to inform changes to a patient’s TP. To test the primary hypothesis that patient outcomes depend on the utilization rate of prescribed ABA treatment dosage (hereinafter, utilization), we performed a retrospective cohort analysis to compare utilization and outcomes for three cohorts of patients diagnosed with mild (*n* = 13), moderate (*n* = 14), and severe (*n* = 9) ASD (corresponding to Levels 1, 2, and 3 of severity for both social communication impairments and RRBs under the DSM-5 diagnostic criteria [7]). The prescribed ABA treatment dosage refers to the BCBA-prescribed number of treatment hours per week; utilization is the percentage of treatment time that is completed per week (in hours) by a patient, relative to the number of weekly treatment hours prescribed by the BCBA for that patient. To test the secondary hypothesis that patients with clinically significant IBs have higher utilization and, consequently, better outcomes, we designed a retrospective cohort study with two cohorts: patients with (*n* = 17) and without (*n* = 19) clinically significant IBs at baseline.

### 2.2. Participant Selection

Patients with an ASD diagnosis per the DSM-5 that received ABA treatment under the pBT Forta model from January 2023 to January 2024 were eligible for inclusion. Patients were referred for ABA treatment by community-based providers after diagnosing ASD using a combination of valid measures across multiple modalities (e.g., rating scales, observation, and clinical interviews). Examples of standardized assessment measures used to evaluate for and establish an ASD diagnosis included Childhood Autism Rating Scale, Second Edition (CARS2), Autism Diagnostic Observation Schedule-Second Edition (ADOS-2), Adaptive Behavior Assessment System-Third Edition (ABAS-3), Checklist for Autism Spectrum Disorder (CASD), and Social Communication Questionnaire (SCQ). Patients in our study cohort were required to have two Vineland-3 assessments performed at least 3 months apart. A total of 36 patients had sufficient data and were included in this study. 

Patient demographic and clinical data were obtained from ABA treatment intake forms completed by the parent and from BCBA-developed TPs (Table 1). For the purpose of the cohort analyses, diagnostic ASD severity level was verified on a case-by-case basis by the study team. Patients were stratified into cohorts based on ASD severity level and the presence of clinically significant IBs at baseline (Supplementary Tables S1 and S2, respectively). IBs were categorized based on the Vineland-3 guidelines that indicate an internalizing (Int) or externalizing (Ext) *v*-scale score of 21–24 for the IB domain (i.e., the Maladaptive Behavior domain) as being clinically significant [42].

### 2.3. Intervention Delivery

To become pBTs, parents received 50 hours of ABA training facilitated by Forta and delivered via an online platform with both synchronous and asynchronous instruction. pBTs were required to pass an Initial Competency Assessment, to demonstrate the skills and knowledge required to deliver ABA treatment to patients under the close supervision of a qualified clinician. The pBTs at Forta have an 86% success rate at passing the Initial Competency Assessment on the first attempt. BCBAs provided supervision and assessed clients via telehealth.

Highly individualized TPs were created within the app by BCBAs in accordance with the standard of care established by the Behavior Analyst Certification Board (BACB) and reflected in the BACB treatment guidelines [23,46]. Each patient was assessed at baseline with a Vineland-3 that was subsequently used by the BCBA in creating their TP. BCBAs conducted functional behavior assessments to inform highly individualized strategies to mitigate these IBs, which identify the IB’s function (e.g., the behavior of elopement could function to avoid an undesirable task) and promote constructive replacement behaviors [47]. TPs additionally included a treatment dosage recommendation made by the BCBA. The pBT Forta model employs a rigorous requirement for BCBAs to facilitate scheduling of supervision sessions with pBTs weekly, or more frequently if clinically indicated, with a minimum of 5% treatment oversight time being provided by a BCBA for each patient. Our BCBAs and clinical directors regularly meet to ensure that each patient is achieving the expected progress.

pBTs delivered ABA during treatment sessions and used the app to record and track patient progress for each ABA session, a process that is described in our prior work [48]. The app also provided pBTs access to the TP to ensure prescribed skill acquisition and behavior reduction goals were addressed during sessions. pBTs recorded data on a trial-by-trial basis on acquisition of target behaviors pursuant to the goals set [48], and these data were graphed to monitor progress. The BCBA monitored patient progress by observing treatment sessions during supervision, as well as by reviewing the graphed pBT-recorded data. TPs underwent ongoing review by the BCBA assigned to monitor that individual patient in the context of patient progress and were modified as clinically necessary. Consequently, standardized assessments were also administered as often as clinically necessary for each patient (e.g., Vineland-3 assessments were administered as often as 3 months apart; mean: 214 days), rather than on a rigid, preset schedule, to ensure that there was no interference in treatment implementation during the course of this study.

### 2.4. Outcome Measurements

Pre- and post-intervention adaptive behavior for each patient was assessed using Vineland-3 scores, including the Adaptive Behavior Composite/ABC (hereinafter, Composite) score, domain scores (i.e., Communication/COMM, Daily Living Skills or Executive Functioning/EF, Socialization/SOC, Motor Skills/MS), and IB *v*-scale scores. The Vineland-3 is an age-standardized metric for assessing adaptive behavior and is commonly used for clinical assessment of individuals on the autism spectrum and for validation of therapeutic interventions [42]. Multiple studies demonstrated that Vineland scores can significantly change during ABA and other types of treatment and can be used to monitor progress, even over shorter time periods (e.g., three months) [15,44,49,50]. Changes in the Composite, COMM, EF, and SOC scores were evaluated relative to previously established minimal clinically important difference (MCID) values, which indicate the minimal score variation needed to create a clinically meaningful change [44,45].

Utilization was retrieved from the treatment information entered in the app by the pBT. Utilization was calculated weekly and then averaged between baseline and follow-up. Utilization was >100% when patients received more treatment than prescribed.

### 2.5. Statistical Analysis

Longitudinal changes in the Vineland-3 scores (Composite, COMM, EF, SOC, MS, Ext IB, Int IB) between baseline and follow-up, baseline Ext IB and Int IB scores, and demographic and clinical data were used to assess patient behavior change as a metric to assess clinical improvement. The change in Vineland-3 scores (Composite, domain- or subdomain-level) was calculated by subtracting the baseline from the follow-up score. A positive change in the Composite, COMM, EF, SOC, or MS score (i.e., score increase) and a negative change in the Ext IB or Int IB score (i.e., score decrease) constitutes improvement. These score changes did not require normalization, given the standardized nature of the Vineland-3, and we report absolute differences in scores. When performing comparisons to literature data, the mean and 95% confidence interval were utilized, as these data were the statistics available. Data from Ostrovsky et al. (2023) and Gerow et al. (2023) were selected for literature comparisons [18,44]. These studies reported changes in Vineland-3 domains in children on the autism spectrum after at least 3 months, but not more than a year, of conventional ABA and caregiver-implemented ABA, respectively. The comparator caregiver (non-pBT)-implemented model provided weekly ABA coaching to caregivers but lacked formalized training and supervision [18]. Dawson et al. (2010), which was focused on an Early Start Denver Model of ABA, was used as a comparator for MS only [43]. Cohen’s *D* was calculated to determine effect size of mean changes in Vineland-3 scores between baseline and follow-up. The significance of the difference in means of variables between the pBT model and comparator models was determined using the Student’s *t*-test, with a *p*-value < 0.05 considered significant. The significance of the difference in medians of variables between pairs of cohorts within the pBT model was determined using the nonparametric Mann–Whitney *U* test, with a *p*-value < 0.05 considered significant. The significance of item-level changes in maladaptive behavior from baseline to follow-up Vineland-3 exam was determined using a paired samples *t*-test, with a *p*-value < 0.05 considered significant.

## 3. Results

After filtering based on the inclusion criteria described above, the analysis cohort contained 36 patients with a mean age of 7.1 years (SD: 3.3 years). Detailed demographics of the full Forta patient cohort are shown in Table 1. Demographic information for Forta patient cohorts stratified for subgroup analyses by severity and clinical significance of IBs are shown in Supplementary Tables S1 and S2. Limited demographic information was available from previously published studies used for comparator cohorts. Ostrovsky et al. (2023) reported a mean patient age of 6 years, 9 months [44]; Dawson et al. (2010) reported that included patients had an age range of 18–30 months [43]; Gerow et al. (2023) reported a population that was >86% male (sex assigned at birth), age < 3–17 years, and race or ethnicity of White (53.3%), Hispanic (26.6%), Black or African American (3.3%), or multiple races (16.6%) [18]. Similar to our study, comparator studies that focused on pediatric populations were selected [18,43,44].

**Figure 1:**
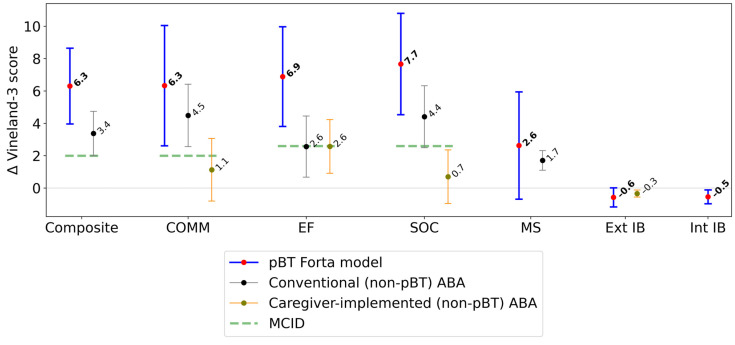
Mean change in Vineland-3 scores (95% confidence intervals), between baseline (pre-pBT-delivered ABA) and follow-up (post-pBT-delivered ABA). Δ = change in Vineland-3 score; Composite = Adaptive Behavior Composite; COMM = communication; EF = executive functioning; SOC = socialization; MS = motor skills; IB = interfering behavior; Ext = externalizing; Int = internalizing; MCID = minimal clinically important difference [44,45]. Improvement is defined as an increase in the Vineland-3 score (positive score change) for Composite, COMM, EF, SOC, and MS; and a decrease in the Vineland-3 score (negative score change) for Ext IB and Int IB.

Patients had a mean prescribed treatment time of 23.5 h/week (SD: 4.6 h/week); mean utilization was 92.2% (SD: 19.5%). Vineland-3 assessments were separated by a mean of 214 days (SD: 54 days). Greater gains from baseline to follow-up assessment were made in the Vineland-3 Composite, and all domain and subdomain scores in the pBT model compared to available data from previously published studies using non-pBT caregiver and conventional (non-pBT) ABA models (Figure 1). As described in the Methods, the comparator caregiver (non-pBT)-implemented model displayed several drawbacks relative to our model [18]. In the pBT model, SOC showed the greatest mean gain, while the Composite, COMM, and EF achieved smaller, but clinically significant, mean score gains. The comparator models and other reports have demonstrated gains in mean score for COMM, EF, and SOC, among other domains and subdomains from ABA-based interventions [18,43,44,45]. The biggest difference in gains was observed between SOC in the pBT Forta model and the comparator caregiver model (7.7 vs. 0.7 points, respectively; Student’s *t*-test: *df* = 50, *t* = 2.82, *p* < 0.01) (Figure 1). EF mean gains for the pBT model vs. the conventional and caregiver models were also high (pBT: 6.9 points vs. comparators: 2.6 points; Student’s *t*-test, pBT vs. conventional: *df* = 212, *t* = 1.92, *p* = 0.06; pBT vs. caregiver: *df* = 66, *t* = 2.34, *p* = 0.02). For both Int and Ext IBs, the pBT model yielded a reduction in mean scores, indicating a reduction in the associated behaviors being evaluated from baseline to follow-up. The reduction in Ext IBs in the pBT Forta model was twice that of the caregiver-implemented ABA model. Data for Int IBs were not available in the literature for comparison. The effect sizes of changes in Vineland-3 Composite scores, domains, and subdomains in the pBT model were modest and ranged between 0.20 (MS) and 0.44 (SOC) (Table 2). This aligns with current literature, where effect sizes for ABA, both with and without parent components, vary between domains [14,27,30].

**Figure 2:**
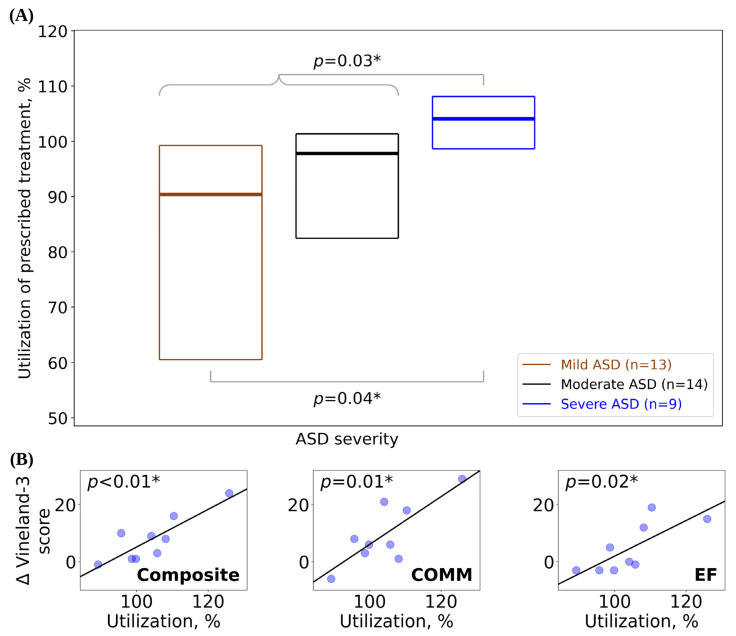
(**A**) Utilization of prescribed treatment (box plots display median as a line between the first and third quartiles) for patients stratified by ASD severity level. (**B**) Change in Vineland-3 scores as a function of utilization for the severe ASD cohort. Best-fit lines generated using simple linear regression (*p*-values for slope ≠ 0, using Wald test with a *t*-distribution). Δ = mean change in Vineland-3 score between baseline and follow-up; Composite = Adaptive Behavior Composite; COMM = communication; EF = executive functioning. * = *p*-value significant at the 0.05 threshold.

Patients were stratified by ASD severity level for evaluation of treatment utilization in order to investigate the relationship between a patient’s ASD symptom and the amount of their weekly prescribed treatment that was used. Utilization was highest (104% median utilization) in the severe ASD cohort (Figure 2A) in comparison to the moderate (98% median utilization) and mild (90% median utilization) ASD cohorts, both of which used a lower absolute number of treatment hours than the severe ASD cohort. Remarkably, the severe ASD cohort had a median utilization above 100%, indicating that pBTs provided a greater number of treatment hours than prescribed. Although utilization was lowest for the mild ASD cohort, this group still showed a high median utilization of approximately 90%. Utilization in the severe ASD cohort was significantly higher than for patients in lower-severity cohorts (Mann–Whitney *U* test, *n* = 9 severe versus *n* = 27 not severe: *U* = 63, *p* = 0.03; *n* = 9 severe versus *n* = 13 mild: *U* = 29, *p* = 0.04). Within the severe ASD cohort, gains in the Composite, COMM, and EF scores showed a significant dose–response relationship with utilization (using a Wald test with a *t*-distribution; Figure 2B). Also within the severe ASD cohort, gains in Int IB scores were significantly greater for patients > 6 years of age at baseline (Mann–Whitney *U* test: *n* = 4 ≤ 6 years of age at baseline, *n* = 5 age > 6 years at baseline, *U* = 1.0, *p* = 0.03); and within the mild ASD cohort, gains in SOC scores were significantly greater for patients > 8 years of age at baseline (Mann–Whitney *U* test: *n* = 9 age ≤ 8 years at baseline, *n* = 5 age > 8 years at baseline, *U* = 31.5, *p* = 0.04).

Within the mild ASD cohort, which showed the greatest variability in utilization, higher utilization was associated with greater reductions in Int IB (Supplementary Figure S1A). When the mild ASD cohort was divided into patients below and above the mean utilization, only one patient from the lower-utilization group (*n =* 6) had a clinically significant Int IB score at baseline, which remained stable at the follow-up assessment. In the higher-utilization group (*n* = 7), five patients had clinically significant Int IB scores at baseline, and three of them improved to non-clinically significant at the follow-up assessment. Patients were also stratified by age (3–5 years; 6–13 years; 14–15 years), diagnosis-to-treatment delay (years to first treatment session after diagnosis), sex assigned at birth (female; male) [51,52,53], and presence of diagnosed psychological comorbidities (attention-deficit/hyperactivity disorder/ADHD, language disorders, anxiety, global developmental delay/GDD) [54] (Supplementary Figure S1B). Greater Int IB improvement was observed in patients seven years of age or younger (Mann–Whitney *U* test: *n* = 21 age ≤ 7 years, *n* = 14 age > 7 years, *U* = 90, *p* = 0.03). A shorter diagnosis-to-treatment delay was also associated with greater Int IB improvement (Mann–Whitney *U* test: *n* = 25 delay ≤ 3 years, *n* = 10 delay > 3 years, *U* = 48.5, *p* < 0.01). Being assigned female sex at birth was the strongest predictive factor for IB improvement, for both Int and Ext IBs (Mann–Whitney *U* test, Int IB: *n* = 8 female, *n* = 27 male, *U* = 176.5, *p* < 0.01; Ext IB: *n* = 8 female, *n* = 27 male, *U* = 148, *p* < 0.01). For both IB Int and IB Ext, individual Vineland-3 items were evaluated for significance of change on the patient-level from baseline to follow-up Vineland-3 exam. IB Int items that patients displayed most often were “Cries or is sad for no clear reason“ (50.0% patients at baseline, 47.1% at follow-up; change over time by paired samples *t*-test: *df* = 34, *t* = 1.44, *p* = 0.16); “Won’t go to/stay at school/work for emotional causes” (44.1% patients at baseline, 32.4% at follow-up; paired samples *t*-test: *df* = 34, *t* = 2.92, *p* < 0.01); and “Lacks interest in things that he enjoys or used to” (35.3% patients at baseline, 17.7% at follow-up; paired samples *t*-test: *df* = 34, *t* = 2.92, *p* < 0.01). IB Ext items that patients displayed most often were “Repeats physical movements over and over” (58.8% patients at baseline, 58.8% at follow-up; paired samples *t*-test: *df* = 34, *t* = 2.09, *p* = 0.04); “Is tricked into doing something that could cause harm” (44.1% patients at baseline, 26.5% at follow-up; paired samples *t*-test: *df* = 34, *t* = 3.69, *p* < 0.01); and “Harms themselves” (32.4% patients at baseline, 29.4% at follow-up; paired samples *t*-test: *df* = 34, *t* = 2.09, *p* = 0.04).

**Figure 3:**
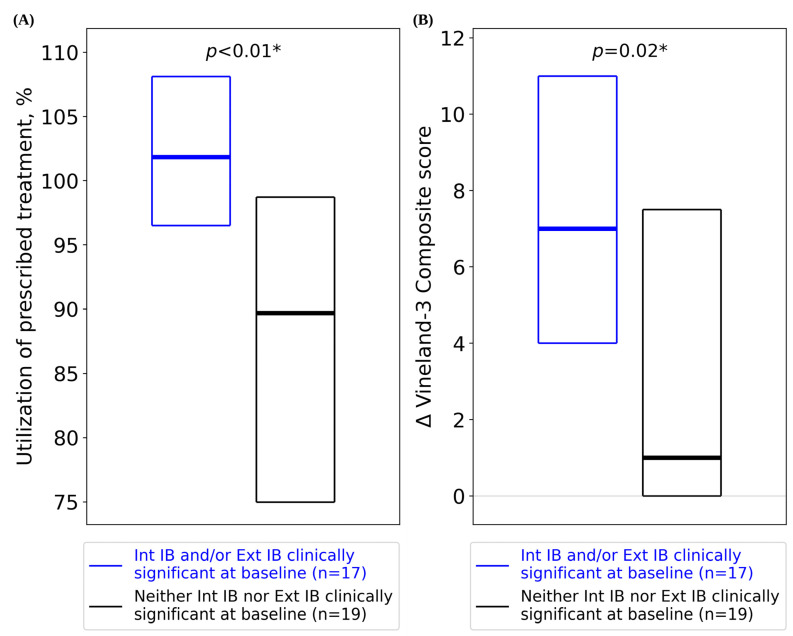
(**A**) Utilization of prescribed treatment hours (box plots display median as a line between the first and third quartiles) for patients with versus without clinically significant IBs at baseline. (**B**) Change in Vineland-3 Composite score (box plots display the median as a line between the first and third quartiles) for patients with versus without clinically significant IBs at baseline. Δ = change in Vineland-3 score between baseline and follow-up; Composite = Adaptive Behavior Composite; IB = interfering behavior; Ext = externalizing; Int = internalizing. * = *p*-value significant at the 0.05 threshold.

Our analysis cohort was further stratified by the presence (*n* = 17) or absence (*n* = 19) of clinically significant Int and/or Ext IBs at baseline. Patients with clinically significant IBs at baseline showed a higher median utilization (>100%) of their prescribed treatment (Figure 3A; Mann–Whitney *U* test: *n* = 17 clinically significant IBs, *n* = 19 non-clinically significant IBs, *U* = 246, *p* < 0.01), similar to the higher median utilization shown by the severe ASD cohort. Patients with clinically significant IBs at baseline also showed greater improvements in Composite score compared to patients without clinically significant IBs (Figure 3B; Mann–Whitney *U* test: *n* = 17 clinically significant IBs, *n* = 19 non-clinically significant IBs, *U* = 237, *p* = 0.02).

## 4. Discussion

In this investigation, we sought to determine if treatment utilization in the pBT Forta model was associated with improved patient outcomes, both with respect to patient baseline and relative to conventional and caregiver ABA models, as measured by Vineland-3. The primary hypothesis, that utilization would be associated with improved treatment outcomes, was supported by statistically significant improvements in multiple Vineland-3 domains and subdomains that were correlated with increased utilization. Additionally, higher utilization was attained by patients with greater ASD severity, with patients with severe ASD showing a dose–response relationship between utilization and Vineland-3 gains. The secondary hypothesis examined how the presence of clinically significant IBs influenced treatment utilization and patient outcomes in the pBT Forta model. Patients with clinically significant IBs utilized more treatment and achieved significantly better improvement in Vineland-3 Composite scores compared to patients without clinically significant IBs. These findings establish that the pBT Forta model performs equal to or better than other ABA models (Figure 1) and is a strong contender to fill the widening treatment accessibility gap. The pBT model overcomes limitations to treatment access [41,48], including logistical challenges (e.g., scheduling, travel to clinical sites) and shortages in the ABA workforce [26,27,28]. The high utilization and clinical gains seen in the pBT Forta model may be driven by multiple factors, including increased accessibility (convenience, geographical proximity, and ongoing consistent operational, technical, and clinical support), a rigorous standard of care, and the confidence and sense of empowerment that parents report they acquire while participating in the pBT model. Parental empowerment has been previously reported with parent ABA training [17,55]; under the pBT Forta model, parents were able to deliver effective care and attain excellent utilization, which may increase this effect. Forta, an ABA provider company, maintains a strong operational framework, including continuous intake, training, and treatment delivery support. Technology is used to streamline the ABA workflow and improve efficiency and consistency; for example, pBTs use our user-friendly app to access the TP and record progress during treatment sessions on any mobile device. For patients with higher ASD severity or clinically significant IBs, enhanced support is required for optimal outcomes [15], and accessing additional treatment time and support is feasible for patients under the pBT Forta model. Statistically significant gains observed in the SOC and COMM domain reflect findings from recent research, indicating that caregiver training and caregiver-led ABA practices can positively impact social and communication skills [33,35] even among children who are non-verbal. Developing these skills is crucial for reducing problematic behaviors [35], for being able to effectively engage in social relationships, and to mitigate any consequences of impaired communication within academic and social situations [33].

Investigating treatment outcomes by payor type indicated equity within the pBT model. Public versus private insurance yielded no differences in Vineland-3 scores (Supplementary Figure S1), similar to results of other ABA providers [44]. This indicates that the pBT model provides all patients with the same quality of treatment. Insurance can offset ABA treatment expenses, and while less than half (21–44%) of individuals on the autism spectrum are covered solely by public insurance [56,57,58], they account for more than half of our patients (Table 1). 

Treatment effectiveness was demonstrated by the correlation between the high utilization in the pBT Forta model and improvements in patient outcomes. Although there are few reports on patient utilization, prior studies indicate that higher utilization may improve outcomes [59] and that >80% utilization is effectively a “full dose” of treatment [15,59,60]. In contrast to Choi et al. (2022), who reported only 28% of patients receiving a full treatment dose [15], the pBT Forta model yielded remarkable utilization rates, with some pBTs delivering more treatment than prescribed (i.e., >100% utilization) (Figure 2A,B and Figure 3A). For each patient, utilization remained substantially unchanged for the study period, which is consistent with our previous findings [41]. The correlation between utilization and improved outcomes suggests that perhaps parental empowerment increases confidence and motivates treatment delivery, and/or the convenient and naturalistic delivery of treatment may play a role in the effectiveness of the pBT model. For example, patients with severe ASD showed higher utilization and Vineland-3 improvements (Figure 2A,B). The high rates of utilization among patients with severe ASD and the corresponding improvements in interfering behaviors may reflect more allocation of treatment time by pBTs to address IBs, which are often more prominent with severe ASD [61,62]. Traditional (non-home-based) ABA treatment often requires travel and time commitments that oftentimes make consistent access to treatment challenging [25]. In contrast, in addition to scheduled treatment time, pBTs can leverage spontaneous, naturalistic ABA techniques within daily interactions, which may account for the increased amount of ABA delivery [63,64]. Naturalistic ABA teaching promotes response generalization, thus increasing the likelihood of skills being incorporated into the repertoire of the child during daily activities [63]. Such approaches are effective, as they help children learn to generalize newly acquired behaviors [65]. Patients with clinically significant IBs showed significantly greater improvements in Vineland-3 Composite scores compared to those who had less severe IBs (Figure 3B), which could be the result of high utilization.

Although higher ASD severity can be associated with increased IB incidence, IBs are variable and pervasive in many individuals on the autism spectrum, including those with less severe ASD [66,67]. In the mild ASD cohort, higher utilization (>90%) resulted in greater improvement in Int IBs (Supplementary Figure S1A). Greater improvement in Int IBs was also observed in patients seven years of age or younger (Supplementary Figure S1B). The types of Int IBs that children experience may change as they mature, and certain conditions intertwined with IBs—such as depression or anxiety—may emerge as children age [68]. Older children may require a longer duration of targeted treatment to address these Int IBs. Int IBs also improved in patients who received treatment within 3 years of diagnosis, which aligns with research indicating that earlier treatment improves outcomes [69]. Patient characteristics assessed for correlations with Vineland-3 changes in our study and the *p*-value of each correlation coefficient are shown in Supplementary Figure S3A and S3B, respectively.

There are several limitations in this study that can be improved with alternative research designs and methods in future work. The primary limitation of this study is the small sample size, resulting from drawing data from Forta patients with the required inputs. This limits the generalizability of our findings. Future work will examine outcomes from a larger number of patients, which will be possible as more caregivers and their children engage in treatment using our pBT model. Another limitation in this study is inter-rater reliability. Though the Vineland-3 is a widely-used assessment metric to measure adaptive behavior in ABA, the quantitative data for each patient was recorded by a single treatment provider (their pBT), with BCBAs performing ongoing supervision and evaluation of patients. Therefore, bias may have been introduced via confounding or intrinsic factors, and the post-test effect for the Vineland-3 is not characterized in the existing literature. Future research may involve clinician adjudication to review data and establish inter-rater reliability. To supplement the utility of the longitudinal data gathered in the pBT Forta model, we compared treatment effects with the available literature data. Although the Vineland-3 is a validated assessment tool developed to establish a quantitative measurement for adaptive behavior, there is the potential for confounding intrinsic or extrinsic factors, which may impact study results from different settings. Additionally, our study used data from patients who had variable lengths of treatment time between assessments; evidence suggests that longer treatment periods lead to better outcomes [70]. Future work should use statistical analysis, such as regression analysis or subgroup analysis, to examine ongoing treatment progress with serial Vineland-3 data to determine the impact of treatment duration on ABA outcomes and the interplay between length of time between Vineland-3 assessments and changes in Vineland-3 scores. Future work could also examine if there are significant differences in Vineland-3 scores when patients are stratified by age within ASD severity. Regarding treatment utilization, it is possible that the high (>100%) utilization rates could have correlated with a need for a higher amount of prescribed ABA hours. High utilization could have also been a function of the convenience of the model, and consistency of ABA delivery. Future work should examine the influence of multiple variables to determine the source of high utilization rates. In addition, more comprehensive assessment of IBs (e.g., Aberrant Behavior Checklist Second Edition/ABC-2) could be performed to gain a more complete understanding of IBs and their change over time, and Vineland-3 IBs could be comprehensively assessed on the item level. Also related to IBs, future work should encompass a subgroup analysis of statistically significant reductions in IBs, and what factors may be contributing to this significance. Finally, with a larger sample size, multivariate statistical analyses could be performed, both to examine the simultaneous effect of multiple variables and to control the effects of variables including time elapsed between Vineland-3 exams.

## 5. Conclusions

Traditional ABA treatment is delivered by behavioral professionals in clinical settings; however, numerous limitations can impair access to this intervention, including ABA provider shortages and financial and logistical barriers. Here we demonstrate the value of our model for rigorous parent-led ABA treatment, which achieved high treatment utilization and improved outcomes within multiple domains and subdomains. Caregiver-led treatment, which can serve as a solution to the numerous barriers associated with accessing ABA, has been highly validated in research. However, to our knowledge, there are no studies examining the clinical outcomes of a formal parent-led ABA treatment program outside of the context of research, wherein caregivers undergo rigorous training in standard of care ABA methods, and become qualified to serve as behavior technicians to provide sustained ABA treatment to their child under the ongoing supervision of qualified clinicians, such as BCBAs. Additionally, there is an overall paucity of research related to factors that impact ABA treatment outcomes. As shown in our results, statistically significant improvement in multiple Vineland-3 domains and subdomains were correlated with increased treatment utilization. Patients with clinically significant IBs utilized more treatment and attained greater gains in Vineland-3 Composite scores compared to patients without clinically significant IBs, which may reflect how convenient access to ABA can increase treatment uptake. A sub-analysis of multiple patient cohorts highlighted factors that may contribute to treatment outcomes, such as age at baseline, delay from diagnosis to treatment initiation, sex assigned at birth, and comorbidities. The findings of this retrospective cohort study affirm that the pBT model can be an effective method for ABA delivery to improve outcomes of individuals on the autism spectrum. The observed clinical outcomes are especially impactful given the robust clinical utility of the pBT model in overcoming existing barriers to care access and filling crucial treatment gaps for individuals on the autism spectrum who struggle to access ABA.

## Figures and Tables

**Table 1:** Patient demographics for the full analysis cohort.

Demographic Category	Category Value	Analysis Cohort (*n* = 36)	Mean of Category (SD)
Patient age at baseline (years)	3–5	15 (41.7%)	7.1 (3.3)
	6–13	19 (52.8%)	
	14–15	2 (5.6%)	
Patient age at diagnosis (years)	2–4	26 (72.2%)	4.2 (2.7)
	5–8	7 (19.4%)	
	9–12	3 (8.3%)	
Diagnosis to 1st session (years)	0–2	18 (50.0%)	3.2 (2.8)
	3–5	12 (33.3%)	
	6–11	6 (16.7%)	
Sex assigned at birth	Male	28 (77.8%)	n/a
	Female	8 (22.2%)	
Payor type	Public	19 (52.8%)	n/a
	Private	17 (47.2%)	
ASD severity level (DSM-5)	Mild	13 (36.1%)	n/a
	Moderate	14 (38.9%)	
	Severe	9 (25.0%)	
Utilization (%)	≤75	15 (41.7%)	92.2 (19.5)
	75–100	11 (30.6%)	
	≥100	10 (27.8%)	
Schooling type	Home	11 (30.6%)	n/a
	Regular	19 (52.8%)	
	Special education	3 (8.3%)	
	None	3 (8.3%)	
Prior/concurrent therapy	Prior ABA therapy	12 (33.3%)	n/a
	Speech therapy	18 (50.0%)	
	Occupational therapy	13 (36.1%)	
	Physical therapy	4 (11.1%)	
Comorbidities	ADHD	9 (25.0%)	n/a
	Language disorders	2 (5.6%)	
	Anxiety	4 (11.1%)	
	GDD	1 (2.8%)	
pBT race/ethnicity	White	16 (44.4%)	n/a
	Black	8 (22.2%)	
	Hispanic/Latino	6 (16.7%)	
	Asian	3 (8.3%)	
	2 or more races	1 (2.8%)	
	Declined to answer	2 (5.6%)	

SD = standard deviation; ASD = autism spectrum disorder; DSM-5 = Diagnostic and Statistical Manual of Mental Disorders, Fifth Edition; ABA = applied behavior analysis; ADHD = attention-deficit/hyperactivity disorder; GDD = global developmental delay; pBT = parent behavior technician.

**Table 2:** Cohen’s *D* calculated for the mean change in Vineland-3 scores, between baseline (pre-pBT-delivered ABA) and follow-up (post-pBT-delivered ABA).

Score Type	Cohen’s *D*
Δ Composite	0.399
Δ COMM	0.282
Δ EF	0.406
Δ SOC	0.435
Δ MS	0.200
Δ Ext IB	0.260
Δ Int IB	0.279

Δ = mean change in Vineland-3 score between baseline and follow-up; pBT = parent behavior technician; Composite = Adaptive Behavior Composite; COMM = communication; EF = executive functioning; SOC = socialization; MS = motor skills; IB = interfering behavior; Ext = externalizing; Int = internalizing.

## Data Availability

The datasets presented in this article are not readily available due to being proprietary. Requests to access the datasets should be directed to the corresponding author.

## Supplementary Materials

The following supporting information can be downloaded at: https://www.mdpi.com/article/10.3390/jcm13082409/s1. The supplementary materials accompanying this manuscript include Supplementary Tables S1 and S2 and Figures S1–S3 reflecting patient demographics and additional results. Supplementary Tables S1 and S2 contain patient demographics for different cohorts. Supplementary Figures S1–S3 reflect additional results, including median Change in Vineland-3 Int IB score (Supplementary Figure S1A,B), within distinct patient cohorts, mean change in Vineland-3 scores by payor type (Supplementary Figure S2), and correlation between changes in Vineland-3 scores and additional variables analyzed in this study and associated *p*-values (Supplementary Figure S3A,B).
