# Supplementary material for: Family-Centric Applied Behavior Analysis Facilitates Improved Treatment Utilization and Outcomes
Source: J Clin Med. 2024 Apr 20;13(8):2409. doi: 10.3390/jcm13082409 (PMC11051390; doi:10.3390/jcm13082409)
Supplement: Supplementary file 1 [file jcm-13-02409-s001.zip › jcm-2950474-supplementary.pdf]

Supplementary Table S1: Demographics for patient cohorts stratified by ASD severity level (DSM-5). ASD = autism spectrum disorder; DSM-5 = Diagnostic and Statistical Manual of Mental Disorders, Fifth Edition; ABA = applied behavior analysis; ADHD = attention-deficit/hyperactivity disorder; GDD = global developmental delay; pBT = parent behavior technician.

**Demographic Category**	**Category Value**	**Mild ASD (n = 13)**	**Moderate ASD (n = 14)**	**Severe ASD (n = 9)**	***P*-value**
Patient age at baseline (years)	3-5	5 (38.5%)	7 (50.0%)	3 (33.3%)	0.43
	6-13	7 (53.8%)	7 (50.0%)	5 (55.6%)	
	14-15	1 (7.7%)	0 (0.0%)	1 (11.1%)	
Patient age at diagnosis (years)	2-4	8 (61.5%)	11 (78.6%)	7 (77.8%)	0.31
	5-8	3 (23.1%)	2 (14.3%)	2 (22.2%)	
	9-12	2 (15.4%)	1 (7.1%)	0 (0.0%)	
Diagnosis to 1st session (years)	0-2	7 (53.8%)	8 (57.1%)	3 (33.3%)	0.50
	3-5	5 (38.5%)	4 (28.6%)	3 (33.3%)	
	6-11	1 (7.7%)	2 (14.3%)	3 (33.3%)	
Sex assigned at birth	Male	11 (84.6%)	11 (78.6%)	6 (66.7%)	0.70
	Female	2 (15.4%)	3 (21.4%)	3 (33.3%)	
Payor type	Public	7 (53.8%)	7 (50.0%)	5 (55.6%)	0.99
	Private	6 (46.2%)	7 (50.0%)	4 (44.4%)	
Utilization (%)	75 or less	4 (30.8%)	2 (14.3%)	0 (0.0%)	0.08
	75-100	6 (46.2%)	8 (57.1%)	4 (44.4%)	
	100 or more	3 (23.1%)	4 (28.6%)	5 (55.6%)	
Schooling type	Home	2 (15.4%)	7 (50.0%)	2 (22.2%)	0.34
	Regular	9 (69.2%)	5 (35.7%)	5 (55.6%)	
	Other or none	2 (15.4%)	2 (14.3%)	2 (22.2%)	
Prior/concurrent therapy	Prior ABA therapy	4 (30.8%)	4 (28.6%)	4 (44.4%)	–
	Speech therapy	8 (61.5%)	6 (42.9%)	4 (44.4%)	
	Occupational therapy	5 (38.5%)	6 (42.9%)	2 (22.2%)	
	Other or none	4 (30.8%)	7 (50.0%)	1 (11.1%)	

Comorbidities	ADHD	4 (30.8%)	4 (28.6%)	1 (11.1%)	–
	Other or none	12 (92.3%)	13 (92.9%)	8 (88.9%)	
pBT race/ethnicity	White	4 (30.8%)	7 (50.0%)	5 (55.6%)	0.58
	Hispanic/Latino	4 (30.8%)	1 (7.1%)	1 (11.1%)	
	Other or declined to answer	5 (38.5%)	6 (42.9%)	3 (33.3%)	

We examined relevant comorbidities (ADHD, language disorders, anxiety, GDD) as binary variables, indicating the presence of at least one or absence of all these four conditions.

Supplementary Table S2: Demographics for patient cohorts stratified by clinical significance of interfering behaviors (IBs) at baseline. ASD = autism spectrum disorder; DSM-5 = Diagnostic and Statistical Manual of Mental Disorders, Fifth Edition; ABA = applied behavior analysis; ADHD = attention-deficit/hyperactivity disorder; GDD = global developmental delay; pBT = parent behavior technician.

**Demographic Category**	**Category Value**	**Clinically significant IBs at baseline (n = 17)**	**No clinically significant IBs at baseline (n = 19)**	***P*-value**
Patient age at baseline (years)	3-5	6 (35.3%)	9 (47.4%)	0.21
	6-13	10 (58.8%)	9 (47.4%)	
	14-15	1 (5.9%)	1 (5.3%)	
Patient age at diagnosis (years)	2-4	11 (64.7%)	15 (78.9%)	0.04*
	5-8	3 (17.6%)	4 (21.1%)	
	9-12	3 (17.6%)	0 (0.0%)	
Diagnosis to 1st session (years)	0-2	7 (41.2%)	11 (57.9%)	0.30
	3-5	6 (35.3%)	6 (31.6%)	
	6-11	4 (23.5%)	2 (10.5%)	
Sex assigned at birth	Male	12 (70.6%)	16 (84.2%)	0.43
	Female	5 (29.4%)	3 (15.8%)	
Payor type	Public	11 (64.7%)	8 (42.1%)	0.18
	Private	6 (35.3%)	11 (57.9%)	
ASD severity level (DSM-5)	Mild	8 (47.1%)	5 (26.3%)	0.05
	Moderate	3 (17.6%)	11 (57.9%)	
	Severe	6 (35.3%)	3 (15.8%)	
Utilization (%)	75 or less	1 (5.9%)	5 (26.3%)	0.01*
	75-100	7 (41.2%)	11 (57.9%)	
	100 or more	9 (52.9%)	3 (15.8%)	
Schooling type	Home	5 (29.4%)	6 (31.6%)	0.75
	Regular	10 (58.8%)	9 (47.4%)	
	Other or none	2 (11.8%)	4 (21.1%)	

Prior/concurrent therapy	Prior ABA therapy	6 (35.3%)	6 (31.6%)	–
	Speech therapy	8 (47.1%)	10 (52.6%)	
	Occupational therapy	6 (35.3%)	7 (36.8%)	
	Other or none	4 (23.5%)	8 (42.1%)	
Comorbidities	ADHD	6 (35.3%)	3 (15.8%)	–
	Language disorders	1 (5.9%)	1 (5.3%)	
	Anxiety	3 (17.6%)	1 (5.3%)	
	Other or none	11 (64.7%)	16 (84.2%)	
pBT race/ethnicity	White	10 (58.8%)	6 (31.6%)	0.35
	Black	2 (11.8%)	6 (31.6%)	
	Hispanic/Latino	2 (11.8%)	4 (21.1%)	
	Other or declined to answer	3 (17.6%)	3 (15.8%)	

Supplementary Fig. S1: (A) Change in Vineland-3 Int IB score (box plots display median as a line between the first and third quartiles) for patients with mild ASD grouped into those below and above the group mean utilization (≤90% versus >90% utilization, respectively). (B) Change in Vineland-3 IB scores (box plots display median as a line between the first and third quartiles) stratified by patient characteristics. From left to right, box plots show the effect of age at baseline, diagnosis to treatment delay, sex assigned at birth, and comorbidities. Δ = change in Vineland-3 score between baseline and follow-up; IB = interfering behavior; Ext = externalizing; Int = internalizing. Improvement is defined as a decrease in the Vineland-3 score (negative score change) for Ext IB and Int IB.

(A)

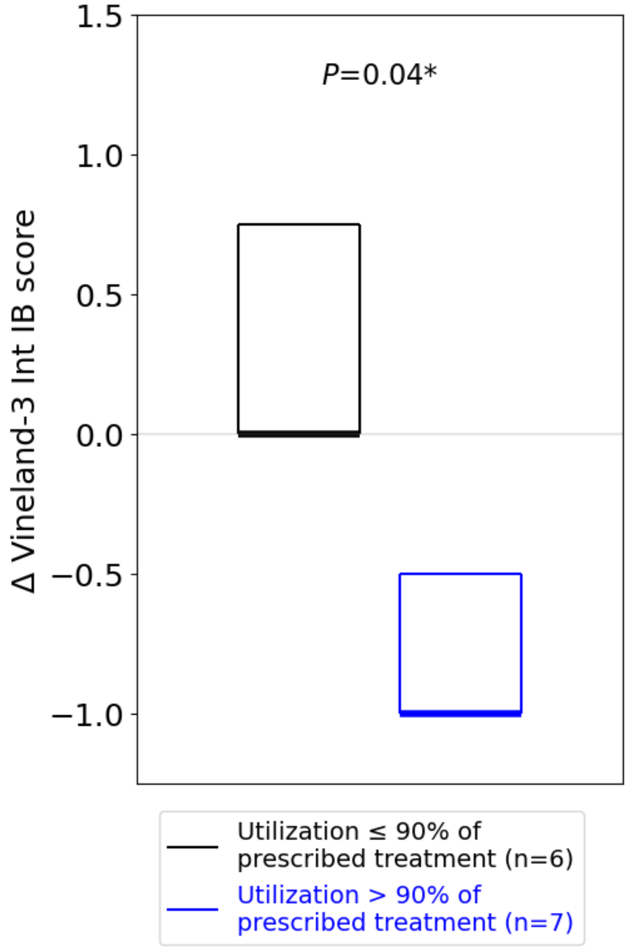

(B)

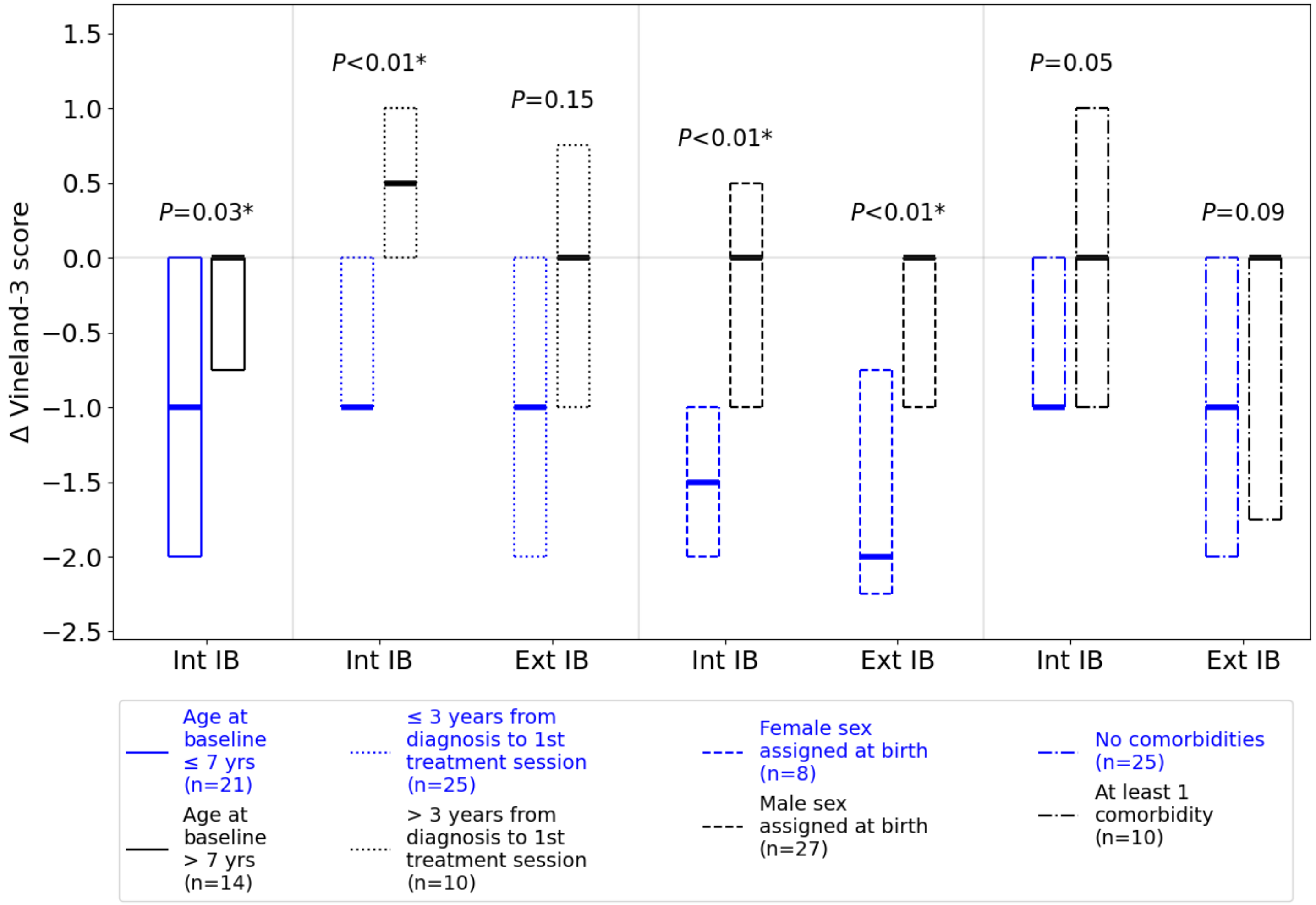

Greater improvement in Int IB was observed in patients who were 7 years of age or younger at treatment initiation compared to older patients (*P*=0.03). A diagnosis-to-treatment delay of ≤3 years was associated with greater improvement in Int IBs (*P*<0.01). Being assigned female sex at birth was the strongest predictive factor for IB improvement, and was significant for both Int and Ext IBs (*P*<0.01). This is a notable validation of the equity achieved within the pBT Forta model, as research suggests that outcomes for females on the autism spectrum can be negatively impacted by IBs (Hartley SL, Sikora DM.; J Autism Dev Disord. 2009 Dec;39(12):1715-22. doi:

10.1007/s10803-009-0810-80); and differences in the types of IBs that impact females vs. males warrant consideration for treatment planning (Aman MG. J Clin Psychiatry. 2005;66 Suppl 10:38-45; and Graziosi G, Perry A, Research in Autism Spectrum Disorders, Volume 100, 2023, 102075, doi.org/10.1016/j.rasd.2022.102075). Despite evidence that comorbidities are predictors of IBs (Casanova MF, Frye RE, Gillberg C, Casanova EL. Front Psychiatry. 2020 Nov 20;11:617395. doi: 10.3389/fpsyt.2020.617395) and may therefore require a higher level of care, comorbidities were not significantly associated with changes to IBs in our model - although they were indicative of potential differences: the mean IB score for patients without comorbidities (n=26) showed improvement, while the mean IB score for patients with comorbidities (n=10) did not change between baseline and follow-up.

Supplementary Fig. S2: Mean change in Vineland-3 scores (95% confidence intervals), between baseline (pre-pBT-delivered ABA) and follow-up (post-pBT-delivered ABA), for patients stratified by type of insurance payor (private or public). pBT = parent behavior technician; ABA = applied behavior analysis; Δ = change in Vineland-3 score; Composite = Adaptive Behavior Composite; COMM = communication; EF = executive functioning; SOC = socialization; MS = motor skills; IB = interfering behavior; Ext = externalizing; Int = internalizing. Improvement is defined as an increase in the Vineland-3 score (positive score change) for Composite, COMM, EF, SOC, and MS; and a decrease in the Vineland-3 score (negative score change) for Ext IB and Int IB.

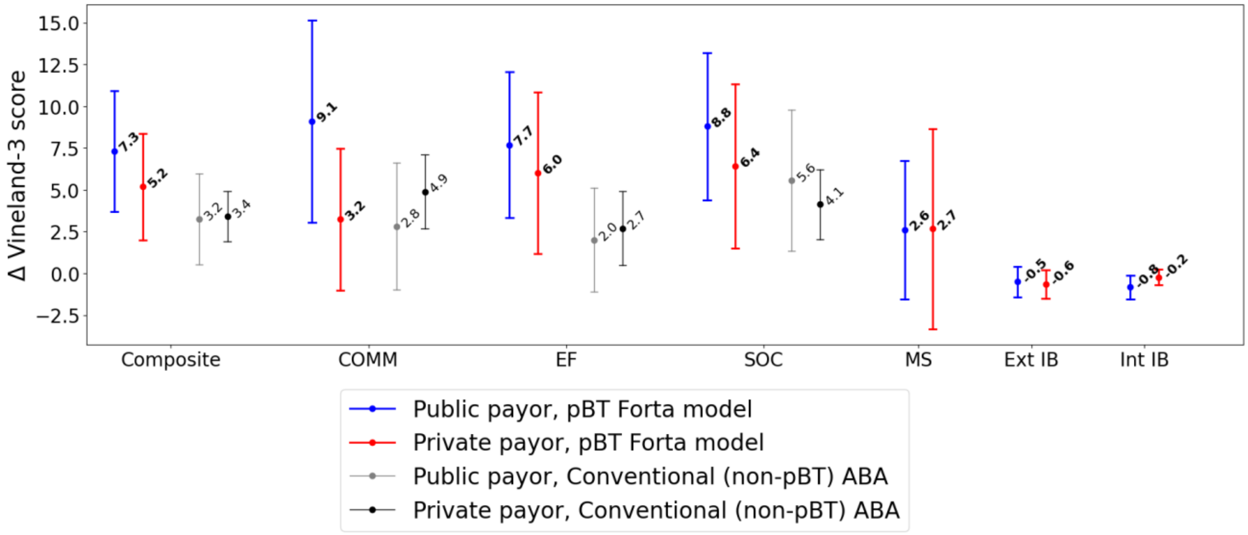

Supplementary Fig. S3: Correlation between changes in Vineland-3 scores, between baseline (pre-pBT-delivered ABA) and follow-up assessment (post-pBT-delivered ABA), and additional variables analyzed in this study. (A) Pearson correlation coefficient (*r*) for each comparison. (B) The *P*-value associated with the respective correlation coefficient for each comparison, computed using the probability density function of *r* (also known as the exact distribution of *r*). A significance threshold of $P<0.05$ was used for classifying a correlation between two variables as significant. pBT = parent behavior technician; ABA = applied behavior analysis; Δ = change in Vineland-3 score; Composite = Adaptive Behavior Composite; COMM = communication; EF = executive functioning; SOC = socialization; MS = motor skills; IB = interfering behavior; Ext = externalizing; Int = internalizing; ASD = autism spectrum disorder; Tx = treatment.

(A)

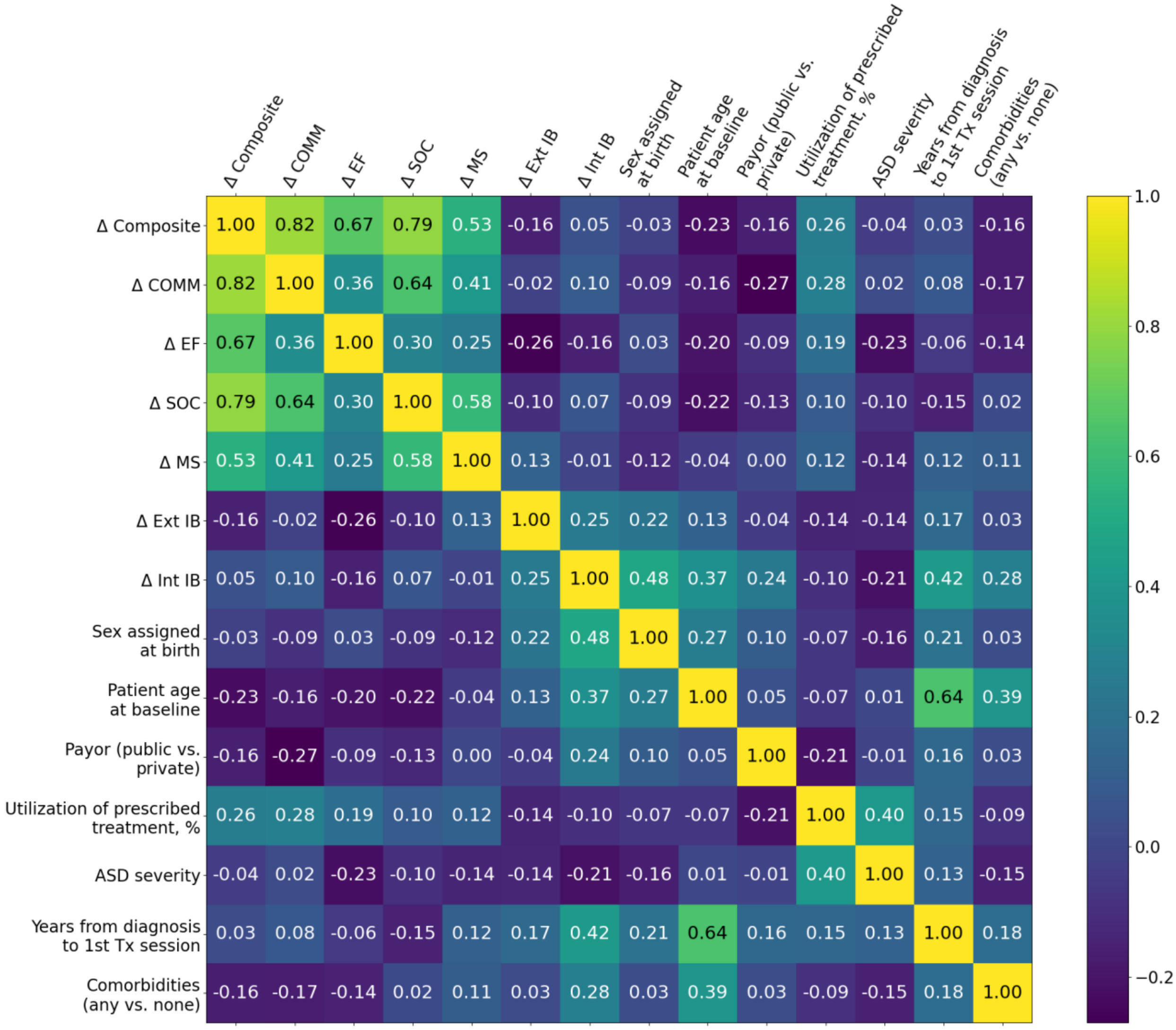

(B)

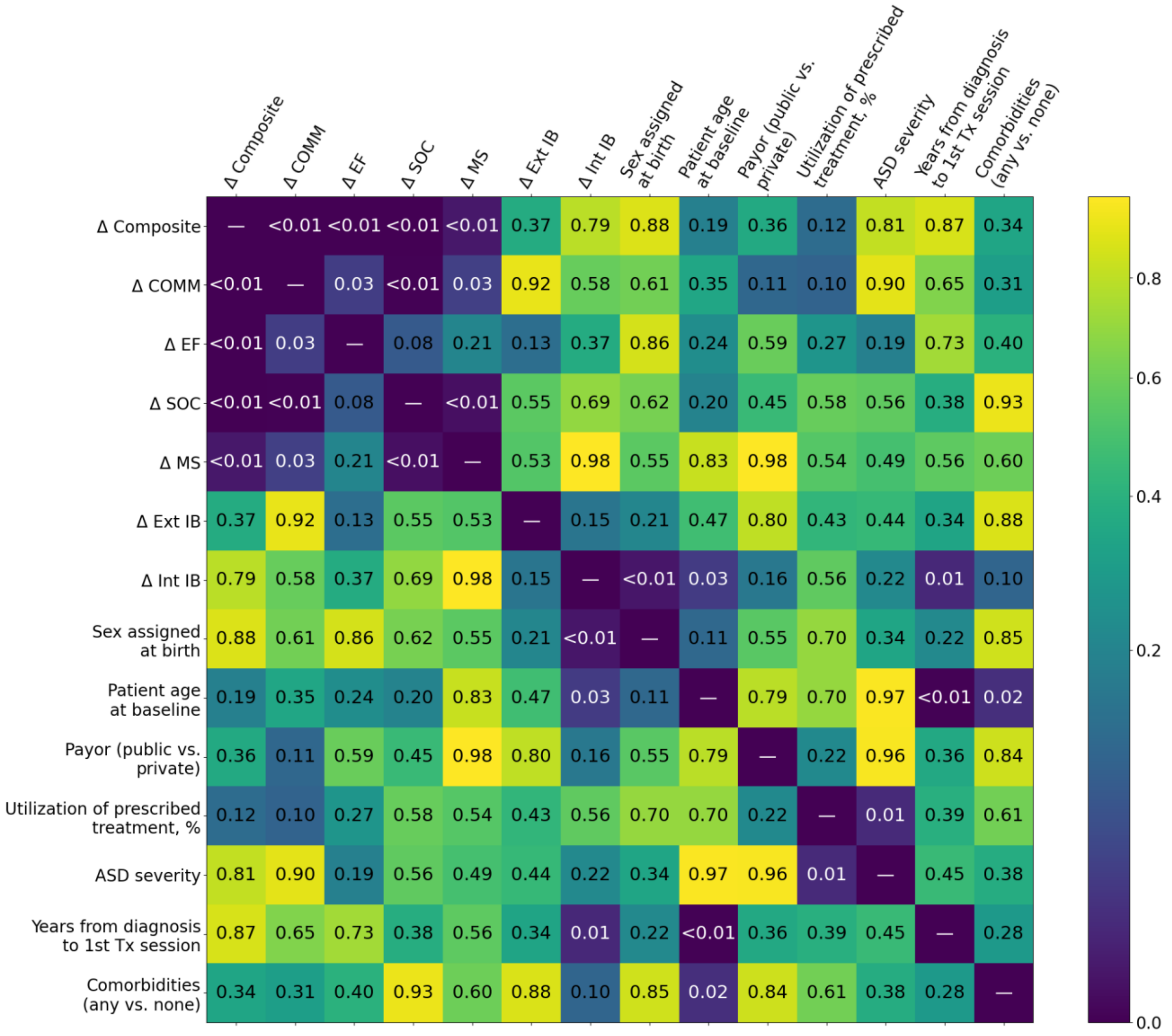
Δ Composite
Δ COMM
Δ EF
Δ SOC
Δ MS
Δ Ext IB
Δ Int IB
Sex assigned at birth
Patient age at baseline
Payor (public vs. private)
Utilization of prescribed treatment, %
ASD severity
Years from diagnosis to 1st Tx session
Comorbidities (any vs. none)
Δ Composite — <0.01 <0.01 <0.01 <0.01 0.37 0.79 0.88 0.19 0.36 0.12 0.81 0.87 0.34
Δ COMM <0.01 — 0.03 <0.01 0.03 0.92 0.58 0.61 0.35 0.11 0.10 0.90 0.65 0.31
Δ EF <0.01 0.03 — 0.08 0.21 0.13 0.37 0.86 0.24 0.59 0.27 0.19 0.73 0.40
Δ SOC <0.01 <0.01 0.08 — <0.01 0.55 0.69 0.62 0.20 0.45 0.58 0.56 0.38 0.93
Δ MS <0.01 0.03 0.21 <0.01 — 0.53 0.98 0.55 0.83 0.98 0.54 0.49 0.56 0.60
Δ Ext IB 0.37 0.92 0.13 0.55 0.53 — 0.15 0.21 0.47 0.80 0.43 0.44 0.34 0.88
Δ Int IB 0.79 0.58 0.37 0.69 0.98 0.15 — <0.01 0.03 0.16 0.56 0.22 0.01 0.10
Sex assigned at birth 0.88 0.61 0.86 0.62 0.55 0.21 <0.01 — 0.11 0.55 0.70 0.34 0.22 0.85
Patient age at baseline 0.19 0.35 0.24 0.20 0.83 0.47 0.03 0.11 — 0.79 0.70 0.97 <0.01 0.02
Payor (public vs. private) 0.36 0.11 0.59 0.45 0.98 0.80 0.16 0.55 0.79 — 0.22 0.96 0.36 0.84
Utilization of prescribed treatment, % 0.12 0.10 0.27 0.58 0.54 0.43 0.56 0.70 0.70 0.22 — 0.01 0.39 0.61
ASD severity 0.81 0.90 0.19 0.56 0.49 0.44 0.22 0.34 0.97 0.96 0.01 — 0.45 0.38
Years from diagnosis to 1st Tx session 0.87 0.65 0.73 0.38 0.56 0.34 0.01 0.22 <0.01 0.36 0.39 0.45 — 0.28
Comorbidities (any vs. none) 0.34 0.31 0.40 0.93 0.60 0.88 0.10 0.85 0.02 0.84 0.61 0.38 0.28 —
0.8
0.6
0.4
0.2
0.0
